# A pure Cutaneous Rosai-Dorfman disease: case report and a review of the literature

**DOI:** 10.22088/cjim.13.4.818

**Published:** 2022

**Authors:** Fatemeh Montazer, Saeedeh Farahani, Zoha Shaka, Zeinab Aryanian, Azadeh Goodarzi

**Affiliations:** 1 Department of Pathology, Iran University of Medical Sciences, Tehran, Iran; 2 Department of Dermatology, Rasool Akram Medical Complex Clinical Research Development Center (RCRDC), School of Medicine, Iran University of Medical Sciences, Tehran, Iran; 3 School of Medicine, Iran University of Medical Sciences, Tehran, Iran; 4 Network of Immunity in Infection, Malignancy and Autoimmunity (NIIMA), Universal Scientific Education and Research Network (USERN), Tehran, Iran; 5 Autoimmune Bullous Diseases Research Center, Tehran University of Medical Sciences, Tehran, Iran; 6 Department of Dermatology, Babol University of Medical Sciences, Babol, Iran

## Abstract

**Background:**

Cutaneous Rosai-Dorfman disease (CRDD) is a rare variant of benign histiocytic proliferative disorder limited to the skin. The underlying etiology is still unclear, but it had been claimed that infections, immunodeficiencies, and autoimmune disorders might have a role in the etiology of this disorder. The characteristic presentation of RDD is lymphadenopathy due to abnormal production and accumulation of histiocytes in lymph nodes; however, the extra-nodal areas could also be affected, such as cutaneous. Herein, we presented a 45-year-old Iranian woman presented with an atypical pure cutaneous Rosai Dorfman disease, in addition to a summarized list of atypical cases of RDD that are reported as pure cutaneous RDD with atypical presentation.

**Case presentation:**

Herein, we presented a 45-year-old woman who referred to us with an ulcerative nodule with a size of 5×5 cm on her buttock, gradually growing over one year. After the primary evaluations, a biopsy specimen was obtained, and histologic studies revealed a dense cellular infiltrate involving the dermis and the subcutis, which was composed of abundant sheets of large histiocytes with admixtures of lymphocytes, plasma cells, neutrophils, and eosinophils within their cytoplasm -known as the emperipolesis phenomenon. The immunohistochemistry staining was positive for S100 and CD68 and negative for CD1a.

**Conclusion:**

The diagnosis of CRDD was confirmed based on these histopathological findings.

Rosai-Dorfman disease (RDD) is a rare disorder first described in 1965 by Destombes, and later in 1969, Rosai and Dorfman recognized it as a distinct entity coined as sinus histiocytosis with massive lymphadenopathy.(1, 2)RDD is classified as the ‘R group ‘of histiocytosis disorders and categorized into three main categories: familial, cutaneous, and sporadic (the most common form)(3)Considering the rareness of the disorder, the exact etiology of RDD remains as yet obscure. However, some studies showed the association between this disorder and viral infections such as the human immunodeficiency viruses (HIV), herpes simplex virus ; and autoimmune disorders like systematic lupus erythematosus (SLE) and Crohn's disease (4, 5). The basic pathology behind RDD is the abnormal accumulation of histiocytes in different tissues and organs; therefore, a wide range of presentations could occur based on the affected area. The isolated cutaneous manifestations without any nodal involvement and systematic features are categorized as CRDD forms of RDD.(6)CRDD is usually presented with painless, non-pruritic, slowly progressive nodules that are purely limited to the dermis and subcutaneous.

A thorough history taking and physical examination, then more evaluations such as excisional biopsy, can help to reach the diagnosis (4, 6). The subacute clinical symptomatology alongside histopathological examinations and immunohistochemical studies will confirm the diagnosis of CRDD. The classic histologic finding in RDD is identified by finding many clusters of histiocyteswith hypochromic nuclei, and phagocytosed lymphocytes, plasma cells, neutrophils within their cytoplasm, and the characteristic immunohistochemical staining analysis for CRDD is reported as positive cytoplasmic and nuclear S100 with CD68 and CD14 accompanied by negative CD1a and CD207 (7, 8). Commonly, RDD is a benign and uncomplicated disease that may have spontaneous remissions; therefore, observation is a reasonable choice of treatment in most cases, but the most effective therapy in the unifocal forms of the disease is surgical excision of the histiocytic lesion (6, 9). Histiocytosis based on its type, may mimic a wide variety of dermatologic disorders, so getting more familiar with different clinical presentations (10, 11) helps us to easier and better approach to the patients. In this regard, reporting different clinical presentations of histiocytosis especially atypical ones are of great value. Herein we present a 45-year-old patient with a solitary nodule growing gradually on the patient's lower extremity without lymphadenopathy or any systematic symptoms.Also, we have a summarized list of atypical cases of RDD that are reported as pure cutaneous RDD with atypical presentation (table1).

**Table 1:** Summary of clinical and pathological information of atypical pure cutaneous Rosai Dorfman disease

**First author/year**	**Sex/ age**	**Presentation**	**Location of lesions**	**Additional findings**	**Immunohistochemistry**	**Histopathology findings**
Mizuta 2021 (21)	M/51	smooth, soft, and mobile red mass size: 2.5 × 2 cm with no surrounding purpura	right temporal region (away from the skull)	-	S100 and CD68: + CD1a, CD34 and ERG: -	Numerous plasma cells in the dermis, many large histiocytes formed a nodular/diffuse pattern
Naqvi 2021 (22)	M/41	asymptomatic 2-cm, erythematous nodular lesion	left shin in the background of a black tattoo	-	CD68 and CD4: + S100: -	Many large, foamy histiocytic cells +emperipolesis
Shen 2021 (23)	M/57	multiple progressive smooth heliotrope plaques or nodule	chest, abdomen, and back	-	CD68 and S-100: + CD1a + in epidermis but - in dermis activated Langerhans cells	Dermoscopy view: yellowish foci and irregularly branching vessels
Tan 2021 (24)	M/55	varying sizes of many dark red nodules and lumps	face, trunk, and limbs	increased level of serum IL-6 and serum IgG4	S100 and CD68, CD38: + CD1a: - Plasma cells showed focally positive for IgG, IgG4 (a ratio of IgG4/IgG >40%)	Diffused dense nodular infiltration of "nude" epithelioid histiocytes with infiltration of minimal lymphocytes and plasma cells around the epithelioid nodules
Bazyluk 2020 (25)	F/52	three gradually enlarging, reddish - brown nodules	right upper extremity	Syphilis +	S100 and CD68: + CD1a: -	Dense dermal polymorphic infiltrate with numerous histiocytes exhibiting emperipolesis
Song 2020 (26)	F/49	pain and swelling of the cartilaginous portion of the ears, swelling of the nasal bridge, and dyspnea	Ears and nose	-	S100: +	Dermal and subcutis infiltration of Pale-staining histiocytes with vacuolated cytoplasm and admixed lymphocytes and plasma cells + emperipolesis
Kim 2019 (27)	M/57	asymptomatic annular-growing brownish patches	both flanks	necrobiotic granuloma in the mid- and deep dermis, consistent with AEGCG	S100: +	Dense histiocytic, lymphoplasmacytic, neutrophilic infiltration in dermis and subcutis +emperipolesis
Lu Gan 2019 (28)	M/47	erythematous papule progressively enlarged to a reddish-purple plaque (without pain or pruritus)	right ear	-	S100 and CD68: + CD1a: -	A dense dermal infiltrates of plasma cells, lymphocytes, pale histiocytes +emperipolesis no epidermal and dermo-epidermal junction changes
Park 2019 (29)	F/70	solitary erythematous plaque with a beaded appearance size: 1.5* 1.5 cm	philtrum	-	PS100, CD68: + CD1a: -	Mixed inflammatory cells in the superficial dermis and the deep dermis, Emperipolesis
Skyes 2019 (30)	M/76	erythematous exophytic nodule size: 10*9 mm	left mid back	-	S100: +	A circumscribed, multilobulated dermal nodular infiltrate with an ulcerated epidermis
Zhang 2019 (31)	M/48	asymptomatic, dome-shaped, red-brown, shiny surfaced papules and nodules	face, limbs and trunk	-	PS100, CD68: + CD1a: -	Numerous infiltrating histocytes +emperipolesis
Lee 2018 (32)	F/49	erythema plaques began as a coin-sized erythematous papule that gradually grew into large, confluent plaques which covered the entire face	right cheek then whole face (giving a leonine appearance)	-	S100 and CD68: + CD1a and CD30: -	Dermal lymphoplasmacytic infiltration admixed with scattered histiocytes
Liu 2018 (32)	M/41	multiple dark-brown erythematous nodules and plaques	bilateral cheeks (the largest plaque on the right cheek), trunk and limbs	-	PS100, CD68: + CD1a: -	Dense infiltration of histiocytes and lymphocytes were seen in the dermis. The histiocytes showed large cytoplasm, vesicular nuclei and evident nucleoli
Yoshimoto 2018 (33)	F/57	subcutaneous mass with indistinct borders size: 5 cm	back (at the site of the previous surgical scar)	-	CD68, CD163 and S-100: + CD1a: -	Dense dermal and subcutaneous infiltration of inflammatory +emperipolesis
Bae 2017 (34)	M/61	multiple exophytic dermal violaceous to hyperpigmented firm, nontender keloidal nodules in a linear distribution	left flank	poorly controlled diabetes hx of laminectomy, a partial cystectomy for a benign bladder mass 10 years ago	PS100, CD68 and CD163: + CD1a: -	Pan dermal sheet-like infiltrate of plasma cells and histiocytes + elastophagocytosis +emperipolesis
Kinio 2017 (35)	F/84	bilateral, erythematous to brown, well-defined, oval to round plaque (atrophy and yellow-gray, well-defined papules also seen)	left chest, face, upper back, and arms	Diagnosed with palindromic arthritis	S100 and CD68: + CD1a: -	Mono- and binucleate histoid cells, xanthoma cells, and neutrophils +emperipolesis
Al-khateeb 2016(6)	M/4	small, itchy, tender, reddish papule size: 1*1 cm to 2.5 cm	skin of the right parotid area	bled at the slightest provocation	S100 and CD68: + CD1a: -	Multinucleated giant cells and eosinophils extending from the reticular dermis to the subcutis
Ciurea 2016 (36)	F/59	hyperpigmentation of the skin overlying the subcutaneous nodules	breast	-	S100: +	Large histiocytes with abundant pale cytoplasm, round vesicular nuclei, lymphocytophagocytosis, or emperipolesis
Kang 2016 (37)	M/45	erythematous mobile nodule without tenderness to palpation size: 1.5* 1 cm	left chin	vascular mass which ultrasonogram of the lesion revealed prominent blood flow	S100 and CD68: + CD1a: -	Infiltration of large histiocytes, lymphocytes, and plasma cells
Kaskas 2015 (38)	F/53	a cluster of 5 erythematous to yellow nodules ranging in size from 0.5 to 1.5 cm	on the midline vertex of the scalp	underlying bony involvement with a small focus of full-thickness calvarial erosion and no intracranial lesion	S100: + CD1a: -	Foam cells, lymphoid aggregates, focally prominent plasma cells, and sparse intermixed granulocytes
Nadal 2015 (39)	F/72	erythematous, infiltrated papules Size: 9 × 6 cm	right breast	-	PS100, CD68: + CD1a: -	Mixed dermal infiltration of lymphocytes, histiocytes and neutrophils with pseudo /true lymphoma a polyclonal and polytypic lymphoid cell and histiocytic population
Yang 2015 (40)	M/52	several asymptomatic red papules with clustered or satellite firm, yellowish-red papules and nodules, that coalesced into plaques exhibiting telangiectasia and an irregular surface	cheeks and forehead	-	S100 and CD68: + CD1a: -	Large histiocytes with vesicular nuclei and abundant pale pink cytoplasm embedded in aggregates of lymphocytes and scattered plasma cells and eosinophils
Fernandez-Vega 2014 (41)	F/51	a superficial soft tissue lesion size: 4 *7 cm in the and papules	left buttock, nose and chin	history of mediastinal mass diagnosed with classical Hodgkin lymphoma, nodular sclerosis subtype 9 years ago	S100, CD11c, CD163, CD33, CD45: + CD302, CD152, PAX-52, CD202, and CD32	Dermal and subcutaneous tissue involvement, sheets of large pale histiocytes on a background of small lymphocytes, neutrophils and plasma cells +Emperipolesis
Han ma 2014 (42)	M/72	erythematous and scaled plaque	right neck	-	PS100, CD68: + CD1a: -	Infiltrate consisting of lymphocytes, histiocytes, sparse population of neutrophils and plasma cells in the dermis
Lin 2014 (43)	M/20s	tender left pinna swelling, multiple pink agminated papules and nodules	left ear (obscuring the external auditory canal)	associated with tinnitus, hearing loss, and headaches	S100: +	Numerous large nodular collections of enlarged, foamy-appearing, lymphocytes, plasma cells, neutrophil and histiocyte +emperipolesis
Potts 2008 (44)	F/31	a solid, 6* 7 cm mass over the of the fixed to the overlying skin, which appeared hyperpigmented	volar radial aspect of right forearm	Exacerbations of crohn’s disease	NA	Subcutaneous infiltrate of histiocytic cells, lymphocytes, plasma cells, and neutrophils histiocytes possess foamy cytoplasm and oval vesicular nuclei with prominent nucleoli. +emperipolesis
Yun-yi Kong 2007 (45)	M/55	Hyperpigmented Infiltrated plaque with brownish papules in the periphery	Right thigh and left calf	-	PS100, CD68: + CD1a: -	Infiltrate comprising lymphocytes, plasma cells, and neutrophils + emperipolesis
Ching-I Lu 2004 (46)	40/F	Dark-red patch Surrounded with papuloplaques	Left thigh	Mild anemia; IgG hyperglobulinemia	CD68 and MAC387: + CD1a: -	NA
(46)	28/M	Dusky-red nodules with confluent plaques	Bilateral cheeks	-	CD68 and MAC387: + CD1a: -	NA
(46)	F/39	Red plaque with nodular surface and central ulceration	nose	Pain of skin lesion; HBV carrier	CD68 and MAC387: + CD1a: -	NA
Ang 1999 (47)	F/51	pustular and acneiform lesions	left side of the abdomen	-	S100 and CD68: +	Histiocytes with abundant pale-staining cytoplasm, Clusters of Russell’s bodies among the plasma cells + emperipolesis
Child 1998 (48)	F/36	Solitary indurated plaque, hyperpigmented skin, numerus firm nodule	Left thigh	High ESR >54 Polyclonal Increase in gamma globulins	S100, CD68: +	Infiltrate of numerous plasma cells in upper and middle dermis, +emperipolesis
Scheel 1997 (49)	M/44	multiple erythematous annular plaques with raised border and central clearing	face, neck, trunk and extremities	-	S-100 and alpha- 1-antichymotrypsin and lysozyme: +	Dermal infiltrate with multiple pale-staining histiocytes and lymphocytes +emperipolesis
Mac-Moune Lai 1994 (50)	M/34	A firm, irregular 2*5 cm mass	inner lower quadrant of the left breast near the areolar margin	-	EMA, Cam 5,2 and AF1/F3: - S100 and CDB8: +	a nodular growth of inflammatory cells of large pale histiocytes, lymphocytes and plasma cells+ sinusal pattern +Emperipolesis
Chu 1992 (51)	M/32	Crusted nodule	scalp	-	CD68 and MAC387: + CDa1: -	NA

**Ethical disclosure**: After describing the rareness of the patient’s condition to her, she orally consented to the authors to use her medical records for publication.

## Case presentation

A 45-year-old woman was referred to our hospital presented with an ulcerative indurated lesion located on her buttock. The lesion started to appear almost a year ago as a single popular lesion which gradually enlarged to a round erythematous nodule with a progressive growth over the last two months. She denied any history of pain, itchy sensations, and secretion from the lesion; also, patient did not experience any constitutional symptom such as malaise, weight loss, and fever over the past year. The patient was in good health and had no significant past medical history or family history of malignancies. 

The physical examination revealed a round reddish-yellow mass approximately 5 cm in diameter located above the right buttock's gluteal fold, and the ulceration was without any evidence of necrosis or bleeding; the rest of her physical examination and her laboratory evaluations were unremarkable. Before this admission, she was treated with antibiotics under the impression of the abscess by another clinician, which did not have any significant effect on her situation. We obtain a biopsy specimen from the lesion, and a histopathological study followed by immunohistochemical (IHC) staining for the patient. The histopathologic analysis reported a dense cellular infiltration involving the dermis and the subcutis with associated sclerosis. 

**Figure1:**
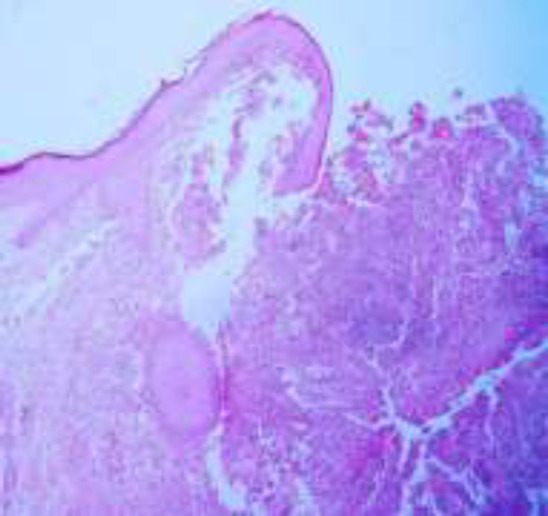
H&E x100: Overlying epidermis shows acanthosis with ulcer

**Figure2:**
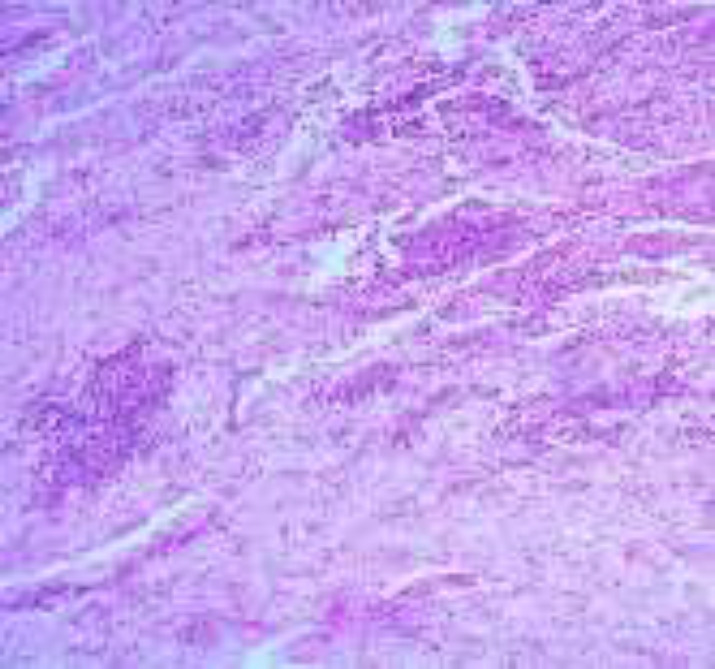
H&E x200: A dense cellular infiltration involving the dermis and the subcutis. The infiltrate is mostly composed of large epithelioid histiocytes with pale eosinophilic cytoplasm admixed with small lymphocytes and plasma cells

**Figure 3:**
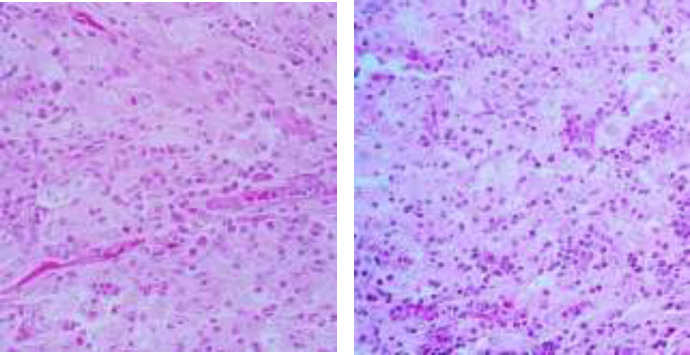
a, bH&E x400: Some histiocytes contain** intracytoplasmic** intact lymphocytes (Emperipolesis)

**Figure 4:**
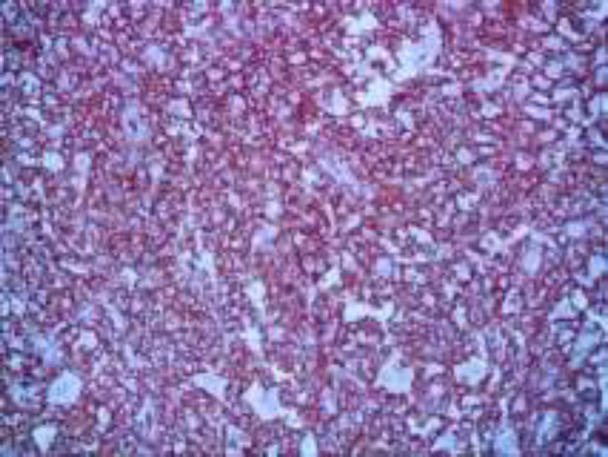
IHC study x 400*: *RDD histiocytes strongly stained with CD45 marker

**Figure 5:**
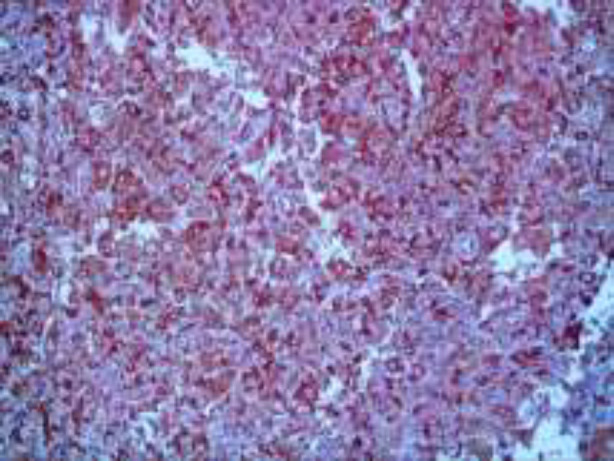
IHC study x 400: Diffuse CD68 cytoplasmic staining of histiocytic population

**Figure 6:**
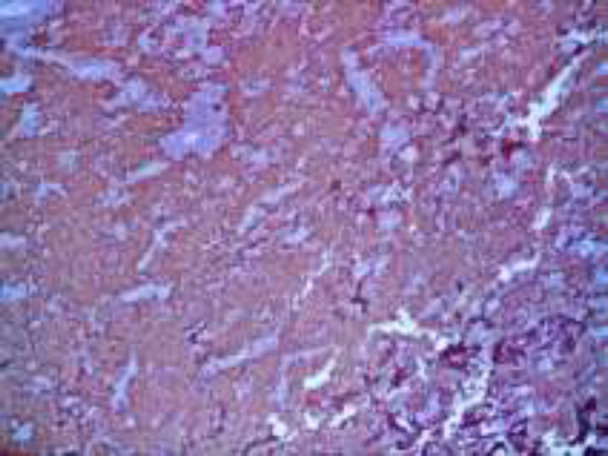
IHC study x 400:***S100 *stain highlights the cytoplasm of RDD histiocytes. S100 negatively outlines intracytoplasmic lymphocytes** in** cells with emperipolesis**

The infiltrate is composed of predominantly of large epithelioid histiocytes with pale eosinophilic cytoplasm. Admixed with these histiocytes are small lymphocytes, plasma cells, neutrophils, and eosinophils (fig 2). Some histiocytes contain intact lymphocytes (emperipolesis) (fig 3a, b). Lymphocytic aggregations in addition to a mild vascular proliferation were also seen. The overlying epidermis shows acanthosis with ulcer (fig1). In IHC, the histiocytes showed expression of S100, CD68 and CD45((figs 4,5,6) and were negative for CD1a.

**Fig 7:**
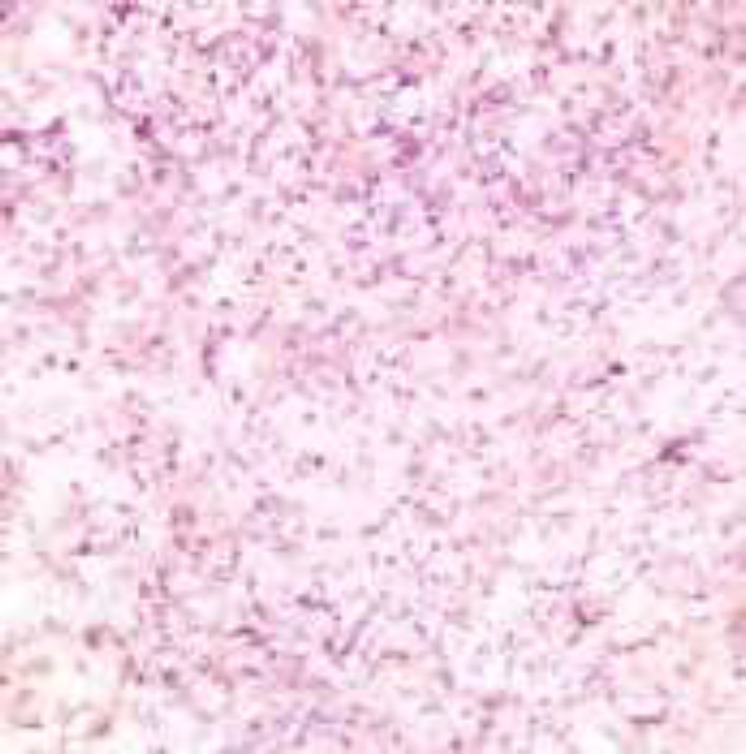
IHC study, x400:  Negative CD1a in histiocytes

## Discussion

The most typical presentation of Rosai Dorfman disease (RDD) is lymphadenopathy; however, extra-nodal sites were also reported to be affected in less than half of the cases.(12)Skin as the most common site is involved in about 10% of RDD cases, but the isolated extra-nodal presentations of RDD are extremely rare -only 3 percent of all reported cases are purely cutaneous RDD.(6)Usual presentations of pure cutaneous RRD, are non-specific dark red or yellow papulonodular lesions or plaques that are commonly located in the face, trunk, and upper and lower limbs.(13) Therefore, our patient presentation is an atypical form of pure CRDD; a solitary ulcerative nodule located on the lower extremity.

From an epidemiological point of view, this disease affects all age groups, sexes, and races, but the demographic pattern and ethnicity distribution differ between CRDD and systemic RDD cases. Systematic RDD is more common in younger patients (mean age of onset 20.6 years) predominantly in men (4:1 ratio) with African ethnicity; on the contrary, CRDD usually affects older individuals (mean age of onset 43.5 years) with a female predominance (2:1 ratio) and is mainly reported in patients with Asian and Caucasian ethnicity (12, 14). Compatible with these patterns, our patient was an Iranian woman whose disease onset was at the age of 44. RDD is known as a benign disorder which rarely has a fatal outcome; patients mostly either have a complete spontaneous remission or have an suitable response to the required treatment (9, 15). The therapeutic strategics are decided based on the patients' clinical circumstances, for example: following and observation in asymptomatic and uncomplicated cases; surgical resection for unifocal extra-nodal manifestations or symptomatic spinal, cranial, or airway involvements; administration of corticosteroids in symptomatic nodal or cutaneous disease; radiotherapy, chemotherapy or immunomodulatory drugs such as thalidomide and rituximab for more severe cases with refractory, disseminated or life-threatening manifestations (16). We chose a surgical approach for our patient considering her unifocal cutaneous manifestation without any systematic involvement or lymphadenopathies. After the nodule excision, she was followed for almost six months, and no refractory or relapsed lesion of RDD was observed, and the patient was symptom-free.

As mentioned, CRDD has a favorable prognosis, but careful evaluations must carry on to distinguish it from its differential diagnoses especially Langerhans cell histiocytosis which is a rare and lethal disease, cutaneous lymphoma and other neoplastic histiocytosis (17). It is essential to perform a comprehensive clinical evaluation along with a thorough immunohistochemical study to diagnose RDD. Some histopathologic features are characteristic for RDD, including typical large histiocytes with contoured hypochromic nuclei and distinct round nucleoli in an abundant, pale, wispy cytoplasm; the presence of emperipolesis phenomenon in histocytes which is the engulfment of intact hematolymphoid and inflammatory cells; and a heterogeneous background of lymphocytes, plasma cells, micro abscess of neutrophils in the absence of eosinophils. Typically, in RDD immunohistochemistry, staining is positive for S100, CD68, CD163, CD14 and negative for CD1a and CD207 (8, 14).

 All these characteristic features, in correlation with clinical presentations, help to distinguish between RDD and other diagnoses, for instance, Langerhans cell histiocytosis, as the most important differential diagnosis for CRDD is positive for CD1a and CD207, has eosinophilic infiltrate, and characteristic features like elongated grooved nuclei and Birbeck granules (18). Other differential diagnoses such as histiocytosis lymphoma, eruptive xanthoma, juvenile xanthogranuloma, and IgG4 related sclerosing disease could also be excluded by immunohistochemical features, clinical and serological evaluations (18, 19). The histopathology of isolated CRDD and systematic CRDD with lymph node involvement are the same, however, some studies suggest CRDD has greater degree of fibrosis, sclerosis, and fewer histocytes with more subtle emperipolesis (18, 20).

In conclusion We presented a patient with an isolated ulcerative dermal-based lesion that had typical cytomorphologic and immunohistochemical features for pure CRDD without any systematic presentation. Thus, the diagnosis of CRDD was confirmed for her condition.
